# HEK293T Cells with TFAM Disruption by CRISPR-Cas9 as a Model for Mitochondrial Regulation

**DOI:** 10.3390/life12010022

**Published:** 2021-12-24

**Authors:** Vanessa Cristina de Oliveira, Kelly Cristine Santos Roballo, Clésio Gomes Mariano Junior, Sarah Ingrid Pinto Santos, Fabiana Fernandes Bressan, Marcos Roberto Chiaratti, Elena J. Tucker, Erica E. Davis, Jean-Paul Concordet, Carlos Eduardo Ambrósio

**Affiliations:** 1 Department of Veterinary Medicine, Faculty of Animal Science and Food Engineering, University of São Paulo, São Paulo 13635-900, Brazil; kroballo@vt.vcom.edu (K.C.S.R.); clesio.gmm@usp.br (C.G.M.J.); sarahingrid@usp.br (S.I.P.S.); fabianabressan@usp.br (F.F.B.); ceambrosio@usp.br (C.E.A.); 2 Edward Via College of Osteopathic Medicine, Blacksburg, VA 24060, USA; 3 Department of Biomedical Science and Pathology, Virginia-Maryland College of Veterinary Medicine, Virginia Tech, Blacksburg, VA 24060, USA; 4 Department of Genetics and Evolution, Federal University of São Carlos, São Carlos 13565-905, Brazil; chiarattimr@gmail.com; 5 Murdoch Children’s Research Institute, Royal Children’s Hospital, Melbourne 3052, Australia; elena.tucker@mcri.edu.au; 6 Department of Paediatrics, University of Melbourne, Melbourne 3010, Australia; 7 Stanley Manne Children’s Research Institute, Ann & Robert H. Lurie Children’s Hospital of Chicago, Chicago, IL 60611, USA; eridavis@luriechildrens.org; 8 Department of Pediatrics, Feinberg School of Medicine, Northwestern University, Chicago, IL 1900, USA; 9 Department of Cell and Developmental Biology, Feinberg School of Medicine, Northwestern University, Chicago, IL 1900, USA; 10 Laboratoire Structure et Instabilité des Génomes, Museum National d’Histoire Naturelle, INSERM U1154, CNRS UMR7196, 75231 Paris, France; jean-paul.concordet@mnhn.fr

**Keywords:** CRISPR-Cas9, gene editing, HEK293T cells, mitochondrial DNA, *TFAM*

## Abstract

The mitochondrial transcription factor A (*TFAM*) is considered a key factor in mitochondrial DNA (mtDNA) copy number. Given that the regulation of active copies of mtDNA is still not fully understood, we investigated the effects of CRISPR-Cas9 gene editing of *TFAM* in human embryonic kidney (HEK) 293T cells on mtDNA copy number. The aim of this study was to generate a new in vitro model by CRISPR-Cas9 system by editing the *TFAM* locus in HEK293T cells. Among the resulting single-cell clones, seven had high mutation rates (67–96%) and showed a decrease in mtDNA copy number compared to control. Cell staining with Mitotracker Red showed a reduction in fluorescence in the edited cells compared to the non-edited cells. Our findings suggest that the mtDNA copy number is directly related to *TFAM* control and its disruption results in interference with mitochondrial stability and maintenance.

## 1. Introduction

*TFAM* is a key mitochondrial modulator with a proposed role in mitochondrial DNA (mtDNA) transcription and replication, and is also required to regulate mtDNA copy numbers [1,2]. *TFAM* is an abundant protein, present in approximately 1000 molecules per mtDNA molecule in mammalian cells, which is enough to coat the entire mitochondrial genome [3]. It has also been shown that *TFAM* is directly involved in mitochondrial genome repair by modulating the access of repair proteins to mtDNA [4,5].

Alterations in mtDNA, such as a decrease in quantity, can cause impaired energy production leading to several clinical disorders. Furthermore, the result of this dysfunction can cause damage to mtDNA compromising mitochondrial function [6,7]. The mtDNA disorders are implicated in many severe diseases caused by molecular defects in oxidative phosphorylation, cell apoptosis, and the lack of a proper bioenergetic supply to mitochondria [8].

The main clinical disorders that are caused by disruptions in mitochondrial DNA are Kearns–Sayre syndrome, LHON, Pearson syndrome, MELAS, MERRF, diabetes, and tumorigenesis ovarian dysfunction [9,10,11].

Disease-related mitochondrial mechanisms are not yet fully elucidated. Considering that *TFAM* plays a crucial role in mitochondrial biogenesis through its interaction with mtDNA, it has emerged as a candidate gene for further investigation [12].

Recent studies have found evidence of putative *TFAM* disorders by linking missense variants in this gene to rare pathologies such as a case of premature death caused by liver failure [13] and a case linked by genomic study to Perrault syndrome, a rare syndrome characterized by premature ovarian insufficiency and hearing loss [14]. A recessive *TFAM* variant report in primary ovarian insufficiency showed that the depletion of *TFAM* can alter mitochondrial function and morphology [8].

*TFAM* has been disrupted previously in mice [15] leading to embryonic lethality. In bovine fibroblasts, editing *TFAM* by CRISPR/Cas9 resulted in a decrease in mitochondrial DNA copy number (mtDNA-CN) and mitochondrial activity [16,17]. 

Gene editing techniques have been used in a variety of applications in human and animal medicine. CRISPR-Cas9 technology has been increasingly applied to the study and treatment of human disease, and the generation of in vitro models is necessary for the understanding and development of therapeutic strategies. Human embryonic kidney 293 (HEK-293) cells are commonly used as hosts for the heterologous expression of proteins and as a general gene-editing model because they have a high transfection efficiency rate and reliably translate and process proteins. This cell line is also quick for reproduction and is easy to maintain in culture. Other characteristics include amenability to transfection using a wide variety of methods and rapid doubling time. Gene-editing constructions tested in HEK 293 cells can easily be adapted to other human cell lineages.

Thereby, the aim of this study was to generate a new in vitro model by CRISPR/Cas9 editing of the *TFAM* locus in HEK293T cells to evaluate the maintenance of mtDNA-CN and confirm its role as an mtDNA regulator in human cells. 

## 2. Materials and Methods

### 2.1. Cell Line

For all experiments, we used the commercial HEK293T cell line (https://www.thermofisher.com/br/en/home/technical-resources/cell-lines/2/cell-lines-detail-153.html, accessed on 15 February 2021). Cells were grown in DMEM medium (Gibco) supplemented with 10% fetal bovine serum (Hyclone), 1% antibiotics-penicillin and streptomycin (Gibco), 50 μg/mL uridine and 100 μg/mL pyruvate in a 5% CO_2_ atmosphere at 37 °C.

### 2.2. CRISPR Design

**Figure 1:**
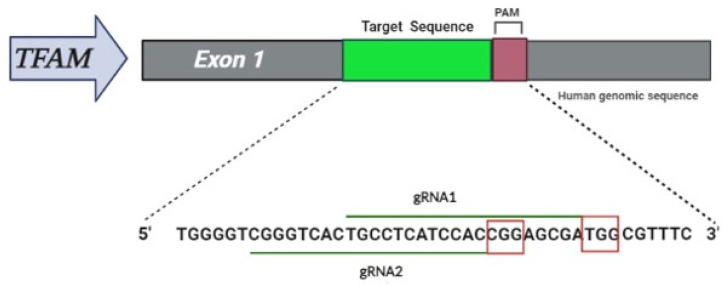
Schematic of the editing strategy, gRNAs designed for *TFAM* exon 1. The target sequence (*TFAM* in gray) is located in exon 1, note in green the target sequence with guides 1 and 2 with their respective protospacer adjacent motifs (PAM)s in red square.

Two gRNAs complementary to *TFAM* exon 1 were designed using the CRISPOR (www.crispor.org, accessed on 10 October 2021) online tool (Figure 1), gRNA1: TGCCTCATCCACCGGAGCGATGG; gRNA2: CGGGTCACTGCCTCATCCACCGG.

We also used a gRNA targeting *ATP1A1* following a strategy reported previously by [18] to improve the post-transfection selection efficiency. To obtain synthetic oligonucleotides coding each gRNA, two complementary oligos were synthesized, annealed, and cloned as described previously by the authors of [16]. The backbone used was pSpCas9(BB)-2A-GFP (PX458) (Addgene #48138) plasmid containing U6-sgRNA and Cas9 expression. 

### 2.3. Transfection Analysis

For the transfection, we used 2 × 10^4^ cells, which were resuspended in Lipofectamine 3000 reagent according to the manufacturer’s instructions. Cells were transfected with 2 μg gRNA (with Cas9-GFP) and 2 μg gRNA *ATP1A1*, pCAG expression vector (a kind gift of M. Jasin, similar to Addgene #26477 but lacking ISceI cDNA) was used as a negative control (2 μg) [18]. After 2 days, cells were analyzed by flow cytometry (BD FACS Melody) to verify the transfection efficiency and then cultivated in the presence and absence of ouabain. After 2 weeks of culture, only cells resistant to treatment with ouabain were still alive.

### 2.4. Genomic DNA Extraction and Analysis of Genome Editing (Insertion–Deletion (Indel) Mutations)

Genomic DNA was extracted from cells using a Qiamp DNA micro kit (Qiagen, Hilden, Germany), according to the manufacturer’s protocol. The genomic region (gRNA target site) was PCR amplified using primers shown in Table 1. PCR products were Sanger sequenced and with these results, it was possible to determine the percentage of indels in the cellular pools by Synthego (https://ice.synthego.com/#/ (accessed on 10 October 2019)) online software tools to evaluate InDels in each region compared to control cells [19]. 

### 2.5. Cell Cloning through Fluorescence-Activated Cell Sorting (FACS) of Transfected Cells

Transfected cells with greater than 70% confluence were isolated by sorting 1 cell/well into 96-well plates using BD FACS Melody. 

These cells were cultured in 100 μL of DMEM supplemented with FBS, uridine, and pyruvate. The cells were incubated at 37 °C with 5% CO_2_ and relative humidity at approximately 80% for 15 days [16]. After in vitro expansion of the selected clones, their mutation rate was confirmed by PCR using *TFAM* primers referring to Table 1 and Sanger sequencing. After sequencing, the single-cell clones were analyzed by tracking indels by Inference of CRISPR Edits (ICE; Synthego, Redwood City, CA, USA; https://ice.synthego.com/#/ accessed on 10 October 2019). This web tool provides an alternative means of analyzing CRISPR gene-editing efficiency using two electropherograms: one from a Cas9-gRNA-treated cell population, and the other from an untreated control or parental cell population, by giving an ICE score (an indel percentage), a KO score (proportion of indels that indicates frameshifts), and an R² regression showing the degree of alignment between the treated and control (parental) cell populations [20].

### 2.6. Determination of mtDNA Copy Number

The mtDNA-CN was estimated as described [21,22]. Briefly, cells were subjected to total DNA extraction using the QIAmp DNA micro kit (Qiagen, 56304), according to the manufacturer’s protocol. The DNA was quantified by spectrophotometry (NanoDrop 2000, Thermo Scientific, Waltham, MA, USA) and frozen at −80 °C. mtDNA quantification was then performed on a real-time PCR thermocycler (Applied Biosystems, 7500 Fast Real-Time PCR System, Foster City, CA, USA) using a commercial assay system (SYBR^®^ Green PCR Master Mix; Life Technologies, Carlsbad, CA, USA) following the manufacturer’s instructions. The samples were analyzed in duplicate using a single-copy nuclear gene (B2M) as control and primers listed in Table 2.

### 2.7. Membrane Potential Using the Mitotracker Red Marker

Cells were cultured in 6-well plates and 0.5 uL/mL of Mitotracker Red (Invitrogen^TM^ —catalog number M22425) was used for cell incubation for 30 min to show mitochondrial membrane potential. Additionally, 1 mg/mL of DAPI (cat# 62248, Thermo Scientific) was added for 5 min to stain the cell nucleus. Photomicrographs were taken with a Zeiss confocal microscope and the images were analyzed by ImageJ software. The ΔΨm was determined considering the estimated red ratio with the appropriate lasers. Corrected total cell fluorescence was measured by ImageJ using integrated density, mean grey, and cell area, which is related to the level of fluorescence present in the cell [17].

### 2.8. Statistical Analysis

One-way analysis of variance (ANOVA) with *p* < 0.01, followed by Tukey’s test and Mann-–Whitney test were used to evaluate the statistical significance the experiments. The statistical analyses were performed using GraphPad Prism 6 (GraphPad Software, Inc., San Diego, CA, USA). 

## 3. Results 

### 3.1. Transfected Cells

**Figure 2:**
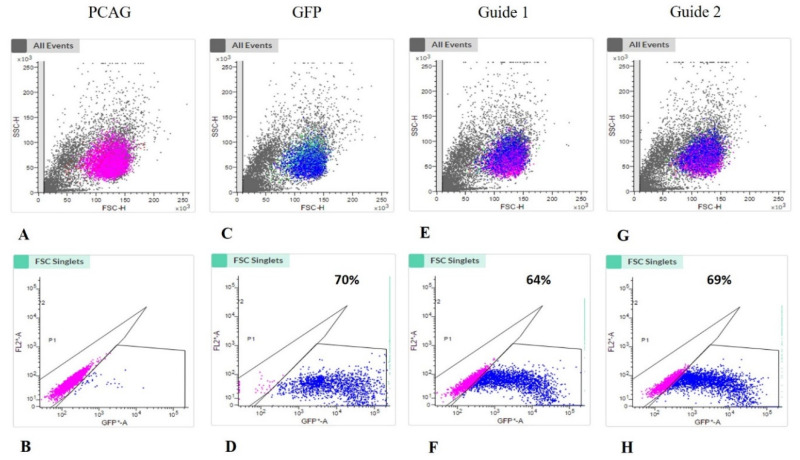
GFP positive immunophenotyping by flow cytometry. In (**A**,**B**) negative control (pCAG) showing sample viability; (**C**,**D**) 70% positive expression of GFP; (**E**,**F**) 64% positive expression of gRNA 1; (**G**,**H**) 69.42% positive expression of gRNA 2.

After transfection, 70% of cells had GFP expression. gRNA 1 and gRNA2 had transfection rates of 64% and 69.4%, respectively (Figure 2). In culture, the cells were treated with 1 mM ouabain to enrich the selection of cells that had undergone genome editing at the *ATP1A1* and *TFAM* loci. 

### 3.2. Editing Efficiency (Cell Pool)

**Figure 3:**
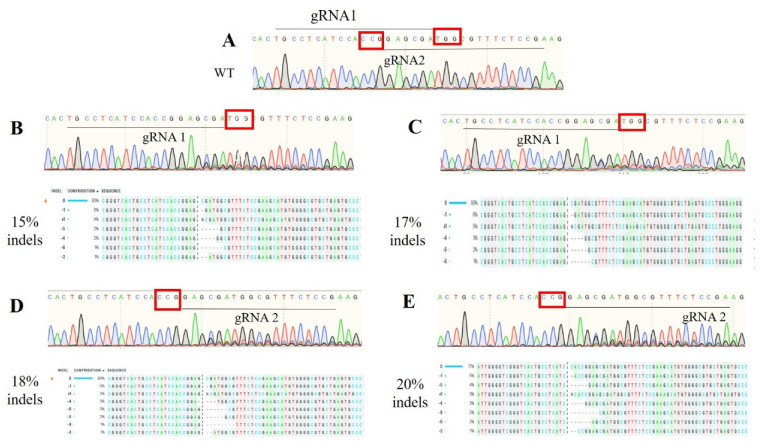
*TFAM* gene mutation detection in cell pools (post-edited cells) before and after treatment with ouabain in culture. In (**A**) the control sequence, note the target region with gRNA2 (underlined), and PAM (red square). In (**B**), before treatment with ouabain note gRNA1 (underlined), PAM (red square), and below the sequences with all possible indels total of 15% and (**C**) after treatment with ouabain note a total of 17% of indels as shown by Synthego ICE. In (**D**) before treatment with ouabain note gRNA2 (underlined), PAM (red square), and below the sequences with all possibilities of indels total of 18% and (**E**) after treatment with ouabain note a total of 20% of indels as shown by Synthego ICE.

Both gRNAs resulted in genome editing of the first exon of *TFAM*. The targeting efficiency was evaluated by PCR amplification (Figure S1), Sanger sequencing, and indel analysis by Synthego ICE tool. The editing efficiencies were 15% and 18% for gRNA1 and gRNA2, respectively, in the cell pool. After co-selection by ouabain in culture, guide 1 improved to 17% and guide 2 to 20% mutation rate (Figure 3). 

### 3.3. Clonal Cell Line Efficiency

**Figure 4:**
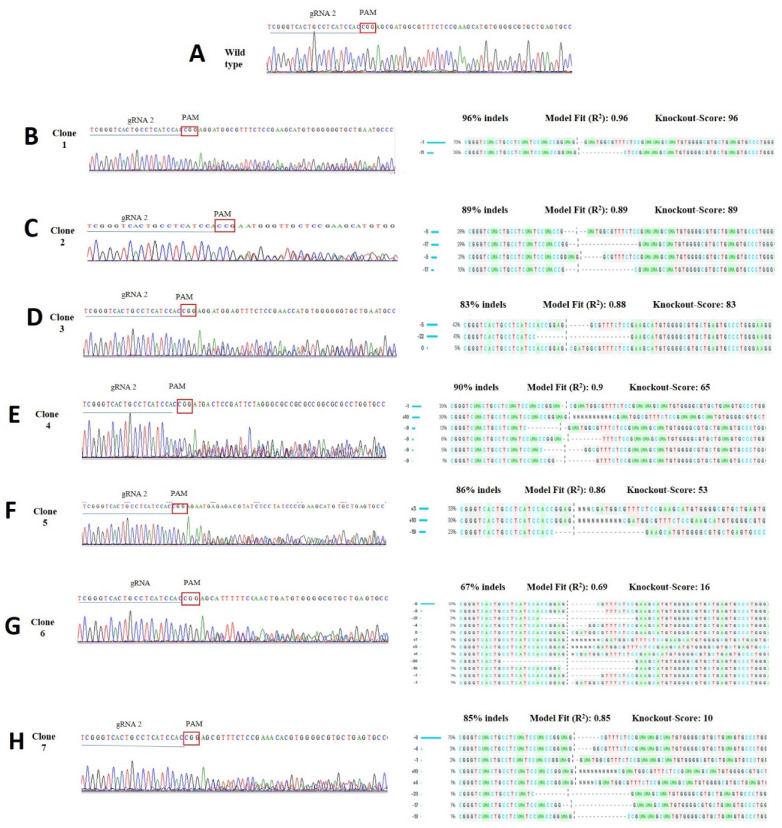
Detection of Cas9/gRNA2-induced mutations of each individual clone. Percentage of indels as shown by Synthego ICE online tool. In (**A**) note the control sequence with gRNA 2 highlighted and the selected PAM (red square). Sequences from B-H are the individual clones, note on the left the target sequence with gRNA 2 and PAM. Note in the target region two alternating peaks demonstrating successful gene editing. (**B**–**H**) sequences on the right side show the mutation rates with the sizes of deletions (−) and insertions (+), with the percentage of indels and KO score for each clone.

The cells were cultured for a period of 15 days and the wells containing individual colonies were selected. From all selected clones, seven showed the band with the expected molecular weight (Figure S2). Clones were Sanger sequenced, and chromatograms were analyzed by the mutation rate (indel) of the clonal population. We obtained seven heterozygous mutant clones with relevant percentages of indels (Figure 4) and Synthego ICE KO score (Table S1).

### 3.4. Determination of mtDNA Copy Number

**Figure 5:**
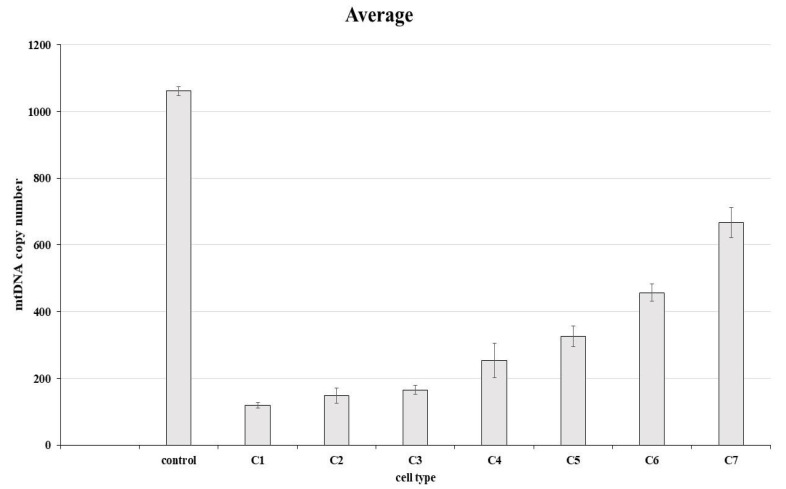
Analysis of mtDNA copy number of edited cells and unedited cells used as control. Note the control and clones 1 to 7. C1–C7. Significant statistical difference (*p* ≤ 0.01).

We observed a decrease (*p* ≤ 0.01) in mitochondrial DNA copy number in the edited clones when compared to unedited ones (control). Unedited clones showed an average of 1061 copies and edited clones (clones 1–7) showed from 118 to 688 copies of mtDNA (Figure 5). Relative comparisons between edited cells (clones 1 to 7) and non-edited cells are found in Table 3. We also noticed that clones with a higher knockout score according to the Synthego ICE tool had a lower mtDNA copy number.

### 3.5. Membrane Potential Using the Mitotracker Red Marker

**Figure 6:**
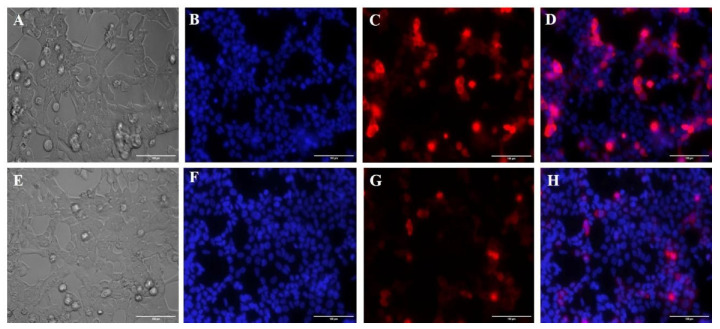
Photomicrograph of epifluorescence with Mitotracker Red marker. Analysis of the mitochondrial membrane potential of edited versus non-edited cells. In (**A**,**E**) control, (**B**–**F**) DAPI marker, (**C**–**G**) Mitotracker Red fluorescence, and (**D**–**H**) merged.

**Figure 7:**
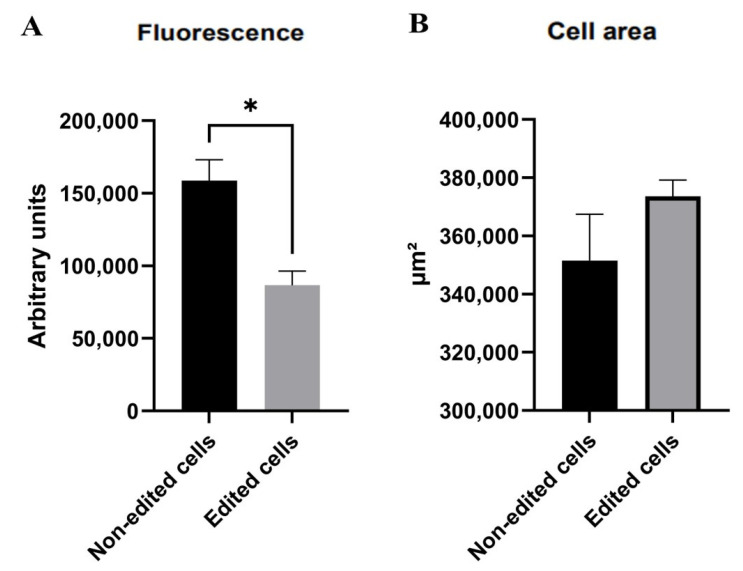
Comparison of Mitotracker Red expression and cell surface area between edited and non-edited cells. (**A**) Red fluorescence intensity in edited and non-edited cells, *p* ≤ 0.05. (**B**) Cell surface area in edited and non-edited cells, *p* ≥ 0.04. * Difference in fluorescence between edited and non-edited cells.

The cell staining with Mitotracker Red shows a reduced red fluorescence in the edited cells (Figure 6). The corrected total cell fluorescence was measured by ImageJ software. When comparing the edited cells with the unedited control, the former showed 54% (on average) less red fluorescence intensity than the latter. When cell surface area was compared no statistical difference was found (Figure 7).

## 4. Discussion

Given the important role of mitochondria in cellular homeostasis, dysfunction of this organelle can lead to several common diseases [23]. The origin of these disorders may be related to variants in genes of the nuclear DNA that encode mitochondrial proteins or directly in the mtDNA [24]. 

Currently, several human pathologies are detected as a result of variants in mtDNA. Several genes are involved in the maintenance of the organelle, among them *TFAM*, which is one of the primary genes responsible for coordinating mtDNA [25].

Even though the knowledge of mtDNA replication and maintenance has increased dramatically in recent years, the understanding of how cellular mtDNA copy number is controlled and maintained is still not fully elucidated [26]. *TFAM* levels are directly related to mtDNA copy numbers with overexpression of human *TFAM* in mice leading to upregulation of mtDNA-CN and knockout of *TFAM* leading to reduced mtDNA content [27]. Several research studies that evaluated a variety of mammalian cell lines from different tissues have reached the same conclusion: reduced expression of *TFAM* leads to reduced mtDNA-CN [28,29,30]. Conversely, overexpression of *TFAM* in the absence of its regulatory protein Lon results in increased mtDNA copy number [31,32]. Even cancer cells show the same pattern, further confirming that *TFAM* levels and mtDNA copy number are directly related [33,34]. 

Our approach in editing *TFAM* by CRISPR-Cas9 in HEK293T cells was aimed to verify the effect of *TFAM* on mtDNA-CN and expand the understanding of mitochondrial dynamics previously established via our study of a bovine model [16,17].

To confirm the indel mutations in our study we used the Synthego ICE (Inference of CRISPR Edits) analysis tool [19]. This web tool is a reliable alternative for analyzing CRISPR gene editing efficiency using two parameters: one from a Cas9-gRNA-treated cell (preferably clonal) population, and the other from the untreated control. ICE gives an ICE score (an indel percentage), a KO score (proportion of indels that indicate frameshift mutations), and an R² showing the degree of alignment between the treated and control (parental) cell populations [20]. In our results, from the eight analyzed clones, two of them had high efficiency in the rate of indels, with a KO-score of 99% and 96% and 5 bp, 1 bp, and 11 bp deletions (putative frameshift mutations). We also noticed that the *TFAM* editing efficiency is directly related to the number of mtDNA copies. The edited clones 1 and 2 had a reduction of ~91.9% and 89.0%, respectively, in mtDNA-CN compared to the control. The other clones that had a lower KO-score showed a smaller reduction in mitochondrial DNA copies, providing evidence that *TFAM* is critical for the maintenance of mtDNA-CN.

Other approaches evaluating this link between *TFAM* disruption and mtDNA content have provided evidence that follows the same pathway. Silencing approximately 90% of *TFAM* gene expression was responsible for up to 75–80% decline in mtDNA copy number of two distinct mammary epithelial cell lines [35]. Another *TFAM* knockdown performed in MKN45 cells revealed that *TFAM* mRNA levels and mtDNA copy number reached a maximum relative decrease at 72 h post-transfection, a decline between 80–90% [36]. 

In a study conducted on zebrafish to understand the impact of *TFAM* in embryogenesis, Otten et al. (2020) blocked up to 80% expression of *TFAM*, which caused a 42% decline in mtDNA-CN. This level of knockdown was sufficient to cause abnormalities in brain, eye, heart, and muscle development. Postnatal developmental disturbances were also observed in two rare cases of a homozygous missense variant in *TFAM*, where an 89% reduction in mtDNA content in the patient’s liver and 79% in muscle led to liver failure and neonatal death [13].

Recently, a research group focused on elucidating how alterations in mtDNA-CN are related to cardiovascular disease generated a stable heterozygous *TFAM* knockout in HEK293T cells and showed a fivefold reduction in the expression levels of *TFAM*, a marked reduction in protein production (>81%), and an 18-fold reduction in mtDNA-CN [37]. In this case, the researchers showed a correlation between low concentrations of mtDNA-CN and abnormal levels of nuclear DNA (nDNA) CpGs methylation at specific sites, which they conclude is one of the mechanisms that may explain how mitochondrial dysfunction affects the onset and resolution of some diseases.

With this same mitoepigenetics approach, some researchers are linking *TFAM* not only to nDNA methylation but also to mitochondrial nucleoid alterations (either by depletion, overexpression, or post-translational modifications) that may be cancer-related [38]. Considering that *TFAM* is a major protein involved in mtDNA packaging and nucleoid formation [39], this also points to it being a key factor in the causality of some diseases.

Cellular mtDNA-CN is relatively stable under normal physiological conditions and alterations in its number can cause pathological changes in tissues and organs. In addition, mtDNA-CN variation has been shown to be associated with cancer development [40], immunosuppression [41], and neurodegenerative diseases [1]. Since many conditions are linked to mitochondrial alterations and considering the lack of reliable measures of mitochondrial dysfunction for clinical practice, researchers are proposing the use of mtDNA-CN as a biomarker for these pathologies [42]. In this context and due to the key role of *TFAM* in mtDNA-CN maintenance this mitochondrial gene arises as a strong candidate in clinical studies. 

Regarding the co-selection strategy, by screening for mutations in *ATP1A1* using ouabain, the percentage of cells with indels at the second locus (in this case *TFAM*) can increase. HEK293T cells were transfected with a CRISPR-dependent base editor (BE3 which is a fusion of mouse APOBEC1, a uracil glycosylase inhibitor (UGI), and the nickase mutant Cas9-D10A) with sgSTOPs targeting the first exon of *SMARCAL1* and the extracellular loop sequence of *ATP1A1* and then cultured in the presence and absence of ouabain [18]. After cultivation, they observed that the number of edited cells increased from 24% to 38.8% in the ouabain-selected group, as determined by RFLP analysis [43]. When replicating the methodology cited above we found that the rate of indels increased but not at the levels described in other studies [43,44]. We hypothesized that more time in culture under the selective conditions could significantly increase the rate of indels in our cell pool.

The Mitotracker Red staining and its fluorescence intensity are correlated with the mitochondrial polarization status, meaning the latter is more emitted in mitochondria with high activity when cultured with the former [45]. Considering that the edited clones had a 54% reduction in fluorescence when compared to control, we show by this decrease in Mitotracker Red staining that *TFAM* disruption directly affected mitochondrial activity. Similar findings were previously shown in bovine after CRISPR-Cas9 *TFAM* targeting and in HEK293 cells modified by siRNA technique (with Cox1 and Cox3 as targets), which also had a decrease in mitochondrial membrane potential [46]. A different approach also showed similar findings, as seen in a study with LRPPRC-silenced cells exhibiting the same fluorescence reduction after Mitotracker staining in HeLa cells after siRNA silencing assay [47].

Another study that investigated the effects of *TFAM* depletion on the morphology and transcriptome of MKN45 gastric cancer cells showed that after the depletion the cells stained with Mitotracker Red presented a reduction of mitochondrial membrane potential, thus confirming that *TFAM* knockdown also causes mitochondrial dysfunction [36].

Human fibroblasts with a recessive missense variant in *TFAM* were also confirmed to possess significant alterations in mitochondrial size and circularity after Mitotracker Red staining [8]. Regarding cell morphology and size, our results did not show statistical differences between edited and non-edited clones.

## 5. Conclusions

Our results suggest that putative *TFAM* heterozygous disruption interferes with mtDNA-CN and mitochondrial membrane potential. This mitochondrial regulation through *TFAM* provides a greater understanding of the organelle’s function and possible pathogenesis. The modulation of mtDNA copy number by manipulating *TFAM* levels may provide a future route to treat not only primary mitochondrial diseases but also to intervene in other human pathologies characterized by mitochondrial impairment.

## Figures and Tables

**Table 1:** Primers used for PCR.

Target Gene	Primer	Sequence (5′–3′)	Product Size
*TFAM*	*TFAM*-f	CCATAGTGCCTCGCTAGTGG	304 bp
	*TFAM*-r	CTACATTCCAACCCCGGGAC	

**Table 2:** Primers used for relative quantification of the target gene (mtDNA) and single-copy nuclear gene (*B2M*).

Target Gene	Primer	Sequence (5′–3′)	Product Size
B_2_M	B_2_M-f	GGCACCCAGCACAATGAAGA	86 bp
	B_2_M-r	GCCAATCCACACGGAGTACTT	
Mt-RNA	MtRNA-f	GCCCTAGAACAGGGCTTAGT	94 bp
	MtRNA-r	GGAGAGGATTTGAATCTCTGG	

**Table 3:** Relative comparison between edited cells (clones 1 to 7) and non-edited cells.

Cell Line	mtDNA Copy Number	% Relative to Control
Control (non-edited)	1061.183333	100
clone 1	118.5933333	11.17557444
clone 2	147.9566667	13.94261124
clone 3	165.53	15.59862418
clone 4	253.5433333	23.89250993
clone 5	325.7766667	30.69937648
clone 6	457.16	43.0802092
clone 7	668.0666667	62.95487742

## Data Availability

Not applicable.

## Supplementary Materials

The following are available online at https://www.mdpi.com/article/10.3390/life12010022/s1, Figure S1: 2.5% agarose gel results from a PCR showing the amplified region containing 304 base pairs from gRNAs 1 and 2., Figure S2: 2.5% agarose gel results from a PCR showing the amplified region containing 304 base pairs from the 8 selected clones, Table S1: Knockout score and mtDNA-CN analysis.
