# Supplementary material for: HEK293T Cells with TFAM Disruption by CRISPR-Cas9 as a Model for Mitochondrial Regulation
Source: Life (Basel). 2021 Dec 24;12(1):22. doi: 10.3390/life12010022 (PMC8779421; doi:10.3390/life12010022)
Supplement: Supplementary file 1 [file life-12-00022-s001.zip › life-1477491-Supplementary.pdf]

life

Supplementary material of HEK293T Cells with TFAM Disruption by CRISPR-Cas9 as a Model for Mitochondrial Regulation

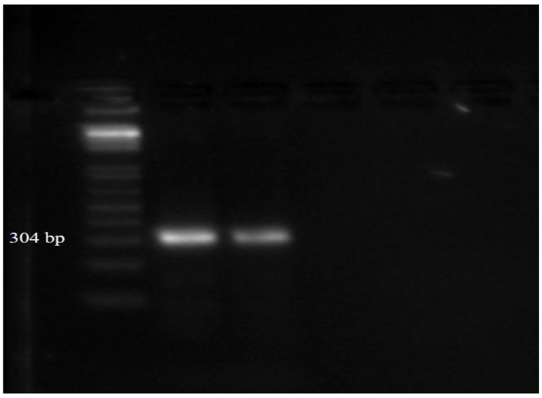

Figure S1. 2.5% agarose gel results from a PCR showing the amplified region containing 304 base pairs from gRNAs 1 and 2.

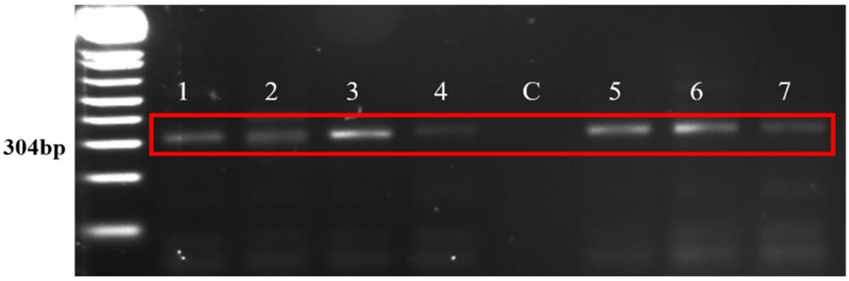

Figure S2. 2.5% agarose gel results from a PCR showing the amplified region containing 304 base pairs from the 8 selected clones.

Table S1. Knockout score and mtDNA-CN analysis.

clones	mtDNA-CN	KO Score %
**clone 1**	119	96
**clone 2**	148	89
**clone 3**	166	83
**clone 4**	254	65
**clone 5**	326	53
**clone 6**	457	16
**clone 7**	668	10
